# Impaired alveolar macrophage 11β-hydroxysteroid dehydrogenase type 1 reductase activity contributes to increased pulmonary inflammation and mortality in sepsis-related ARDS

**DOI:** 10.3389/fimmu.2023.1159831

**Published:** 2023-04-27

**Authors:** Rahul Y. Mahida, Siân Lax, Christopher R. Bassford, Aaron Scott, Dhruv Parekh, Rowan S. Hardy, Babu Naidu, Michael A. Matthay, Paul M. Stewart, Mark C. Cooper, Gavin D. Perkins, David R. Thickett

**Affiliations:** ^1^ Birmingham Acute Care Research Group, Institute of Inflammation and Ageing, University of Birmingham, Birmingham, United Kingdom; ^2^ Institute of Cancer and Genomic Sciences, University of Birmingham, Birmingham, United Kingdom; ^3^ Department of General Critical Care, University Hospitals Coventry and Warwickshire NHS Trust, Coventry, United Kingdom; ^4^ Institute of Clinical Sciences, University of Birmingham, Birmingham, United Kingdom; ^5^ Cardiovascular Research Institute, Department of Medicine, and Department of Anaesthesia, University of California San Francisco, San Francisco, California, CA, United States; ^6^ School of Medicine, University of Leeds, Leeds, United Kingdom; ^7^ Faculty of Medicine and Health, University of Sydney, Sydney, NSW, Australia; ^8^ Warwick Medical School, University of Warwick, Warwick, United Kingdom

**Keywords:** alveolar macrophage (AM), ARDS (acute respiratory disease syndrome), sepsis, 11b-hydroxysteroid dehydrogenase type-1, autocrine action

## Abstract

**Background:**

Acute Respiratory Distress Syndrome (ARDS) is a devastating pulmonary inflammatory disorder, commonly precipitated by sepsis. Glucocorticoids are immunomodulatory steroids that can suppress inflammation. Their anti-inflammatory properties within tissues are influenced by their pre-receptor metabolism and amplification from inactive precursors by 11β-hydroxysteroid dehydrogenase type-1 (HSD-1). We hypothesised that in sepsis-related ARDS, alveolar macrophage (AM) HSD-1 activity and glucocorticoid activation are impaired, and associated with greater inflammatory injury and worse outcomes.

**Methods:**

We analysed broncho-alveolar lavage (BAL) and circulating glucocorticoid levels, AM HSD-1 reductase activity and Receptor for Advanced Glycation End-products (RAGE) levels in two cohorts of critically ill sepsis patients, with and without ARDS. AM HSD-1 reductase activity was also measured in lobectomy patients. We assessed inflammatory injury parameters in models of lung injury and sepsis in HSD-1 knockout (KO) and wild type (WT) mice.

**Results:**

No difference in serum and BAL cortisol: cortisone ratios are shown between sepsis patients with and without ARDS. Across all sepsis patients, there is no association between BAL cortisol: cortisone ratio and 30-day mortality. However, AM HSD-1 reductase activity is impaired in patients with sepsis-related ARDS, compared to sepsis patients without ARDS and lobectomy patients (0.075 v 0.882 v 0.967 pM/hr/10^6^ AMs, p=0.004). Across all sepsis patients (with and without ARDS), impaired AM HSD-1 reductase activity is associated with defective efferocytosis (r=0.804, p=0.008) and increased 30-day mortality. AM HSD-1 reductase activity negatively correlates with BAL RAGE in sepsis patients with ARDS (r=-0.427, p=0.017). Following intra-tracheal lipopolysaccharide (IT-LPS) injury, HSD-1 KO mice demonstrate increased alveolar neutrophil infiltration, apoptotic neutrophil accumulation, alveolar protein permeability and BAL RAGE concentrations compared to WT mice. Caecal Ligation and Puncture (CLP) injury in HSD-1 KO mice results in greater peritoneal apoptotic neutrophil accumulation compared to WT mice.

**Conclusions:**

AM HSD-1 reductase activity does not shape total BAL and serum cortisol: cortisone ratios, however impaired HSD-1 autocrine signalling renders AMs insensitive to the anti-inflammatory effects of local glucocorticoids. This contributes to the decreased efferocytosis, increased BAL RAGE concentrations and mortality seen in sepsis-related ARDS. Upregulation of alveolar HSD-1 activity could restore AM function and improve clinical outcomes in these patients.

## Introduction

Acute Respiratory Distress Syndrome (ARDS) is a devastating inflammatory disorder of the lungs, with sepsis being the most common precipitating factor. Inflammation is the appropriate physiological response to infection, associated with both the innate and adaptive immune responses, thereby aiding defence against pathogens. Normally, homeostatic mechanisms including activation of the hypothalamic-pituitary-adrenal axis leading to increased anti-inflammatory endogenous cortisol release, promote resolution of inflammation after the infective agent has been cleared. Serum free cortisol levels are elevated in patients with critical illness (1, 2). However, in ARDS an exaggerated persistent inflammatory response occurs despite increased endogenous glucocorticoid release, which accounts for some of the tissue injury.

Glucocorticoids represent a class of endogenous and synthetic steroid hormones that possess potent anti-inflammatory and immunomodulatory properties (3). Their anti-inflammatory properties have been shown to be significantly influenced by their pre-receptor metabolism by the 11β-hydroxysteroid dehydrogenase (HSD) enzyme in peripheral tissues (4, 5). HSD has two isozymes: The type 1 isozyme of HSD (HSD-1) predominantly acts as an oxo-reductase *in vivo* to convert inactive cortisone to active cortisol (6). HSD-1 thereby locally amplifies glucocorticoid action by generating active cortisol from cortisone within specific cells and tissues. HSD-1 knockout mice show greater cellular infiltration and acute inflammatory injury in models of inflammatory arthritis, sterile peritonitis and pleurisy (7, 8).

Specific tissues throughout the body express HSD-1, including the liver, adipose tissue, bone, eyes, and lungs (9). HSD-1 expression is induced when human monocytes differentiate into tissue macrophages (10). At a systemic level, the ratio of cortisol: cortisone can be an indicator of HSD enzymatic activity. Previous studies have shown that plasma cortisol: cortisone ratios are elevated in critically ill patients with sepsis, indicating an increase in systemic HSD-1 activity (11). However, there is a dearth of information regarding cortisol metabolism in the lung and specifically within alveolar cells in critically ill patients with ARDS.

HSD enzymes contribute to autocrine (same cell), paracrine (neighbouring cells) and endocrine (distant cells) glucocorticoid signalling. The importance of autocrine HSD-1 signalling in modulating macrophage function has recently been demonstrated in a murine model of inflammatory polyarthritis (12). Myeloid-specific deletion of HSD-1 diminished the anti-inflammatory response of macrophages to glucocorticoid treatment, leading to greater local joint inflammatory injury and destruction. However, the circulating glucocorticoid levels were unchanged compared to wild type mice (12). Myeloid-specific HSD-1 deletion has also been associated with promotion of inflammatory angiogenesis in similar models (13). Thus, local HSD-1 expression in macrophages plays an important role mediating the anti-inflammatory effects of glucocorticoids at peripheral sites of inflammation.

We have previously reported that alveolar macrophage (AM) efferocytosis (clearance of apoptotic cells) is impaired in patients with sepsis-related ARDS, and is associated with increased mortality (14). Efferocytosis is a process associated with the suppression of inflammatory responses and promotion of tissue repair (15, 16). Glucocorticoids have the ability to upregulate macrophage efferocytosis; but this capacity is dependent on HSD-1 expression (17, 18). Murine studies have shown that macrophage HSD-1 deficiency is associated with impaired efferocytosis of apoptotic neutrophils in a model of peritonitis (18). Thus, the presence of HSD-1 contributes to the ability of glucocorticoids to induce a pro-resolving macrophage phenotype (19).

We hypothesised that in sepsis-related ARDS, AM HSD-1 activity is impaired, leading to dysregulated autocrine steroid signalling, defective efferocytosis, increased inflammation and greater mortality. To address this hypothesis, we have collated data across two observational studies of critically ill patients with sepsis and ARDS (all recruited prior to the COVID-19 pandemic), and also from models of inflammatory lung injury and sepsis in HSD-1 knockout mice.

## Methods

### Clinical studies

We have combined data from two clinical cohorts of invasively ventilated sepsis patients with and without ARDS.

A bronchoscopic sub-study of the BALTI-2 clinical trial (20) was conducted between December 2006 and March 2010 at the intensive care units (ICUs) of Birmingham Heartlands Hospital and Queen Elizabeth Hospital Birmingham, U.K. Ethical Approval was obtained prior to commencement of the study (REC 06/Q1604/123). Twenty adult patients with sepsis-related ARDS were recruited; of these, serum samples were collected from 20 patients and broncho-alveolar lavage (BAL) fluid samples collected from 19 patients. Five adult ventilated sepsis patients without ARDS were recruited as a control group; BAL and serum were collected from all 5 patients. ARDS was defined as per American-European Consensus Conference (AECC) criteria (21) and sepsis was defined as per the 2001 definition criteria (22). Eligible participants were invasively ventilated adults, within 72 hours of ARDS onset (or meeting sepsis criteria for controls). Exclusion criteria included pregnancy, treatment with intravenous or aerosolised β-2 agonists, treatment with β-adrenergic antagonists, imminent withdrawal of medical treatment, chronic liver disease, and enrolment in another clinical trial within the previous 28 days. Alveolar macrophage HSD-1 activity was measured in all patients from whom BAL was collected.

The AM-ARDS study was conducted at the ICU of Queen Elizabeth Hospital Birmingham, U.K. from December 2016 to January 2019. Ethical approval was obtained to recruit invasively ventilated adult sepsis patients, with and without ARDS (REC 16/WA/0169). Sepsis was defined according to Sepsis-3 criteria (23). Patients who fulfilled the Berlin criteria (24) within the previous 48 hours were classified as having ARDS, whereas those without ARDS were defined as controls. Exclusion criteria included imminent treatment withdrawal, steroid therapy prior to admission, abnormal clotting precluding bronchoscopy, and clinically relevant immunosuppression. In the AM-ARDS study, 38 patients were recruited, 21 with ARDS and 17 without ARDS (controls). Of those with ARDS, 21 had serum collected and 17 had BAL collected, with AM HSD-1 activity measured in 7. Of those without ARDS, 16 had serum collected, 14 had BAL collected, with AM HSD-1 activity measured in 7. See **Supplemental Figure S1** for details of patient recruitment and sampling.

For both studies, samples were collected within 48 hours of initiation of mechanical ventilation. For patients without capacity, permission to enrol was obtained from a legal representative. In both studies, patients were unable to give informed consent due to alterations in conscious level caused by illness and therapeutic sedation. Therefore, their next of kin were requested to give assent for the patient to be recruited into the study. Bronchoscopy, collection of blood and BAL, AM isolation, assessment of AM HSD-1 activity and quantification of cytokines and steroids within biofluids was performed using the same methodology and kits across both studies. Samples from the BALTI-2 sub-study were all collected and analysed in the period 2006 - 2010. Samples from the AM-ARDS study were collected and analysed in the period 2016 – 2019. Blood samples were also collected from 4 healthy volunteers and analysed in 2019.

### Human blood and broncho-alveolar fluid collection

Blood was collected by peripheral venepuncture or from pre-sited arterial lines into Lithium Heparin and Serum clot activator vacutainer tubes (Becton Dickinson Ltd, Oxford, UK). Samples were then transported to the laboratory for processing and analysis. Blood in serum clot activator tubes was allowed to clot at room temperature for 30 minutes. All blood samples were centrifuged at 560 g for 10 minutes at 4°C (Eppendorf AG 5810R centrifuge, Germany). Serum and plasma were aspirated and stored at -80°C.

Bronchoscopy and BAL fluid collection was performed on sedated, mechanically ventilated patients using an identical standardised protocol as part of both the BALTI-2 sub-study and the AM-ARDS study. Bronchoscopy was performed within 48 hours of initiation of mechanical ventilation, and only if the consultant ICU physician responsible for the patient’s clinical care agreed that it would be safe to proceed. Patients were ventilated using 100% inspired oxygen for 5 minutes prior to bronchoscopy. An Olympus LF-TP fiberoptic scope (Olympus-Keymed, UK) was inserted through the patient’s endo-tracheal tube or tracheostomy tube, and the tip was wedged into a sub-segmental bronchus of the lingula or right middle lobe. Three 50 ml aliquots of sterile 0.9% saline at room temperature were instilled as a lavage, and the BAL fluid was aspirated. The sample was immediately placed on ice and transported to the laboratory for processing. BAL fluid was first filtered through sterile surgical gauze to remove mucus. Cell viability was assessed using trypan blue. Differential cell count was performed using cytospin and Diff-Quik labelling (Gentaur Europe, Kampenhout, Belgium). The filtered BAL was then centrifuged at 560 g for 10 minutes at 4°C. Acellular BAL supernatant was aspirated and stored in 1 ml aliquots at -80°C for future analysis. The cell pellet was re-suspended in 10 ml of RPMI 1640 media (Sigma-Aldrich, UK) containing 10% Fetal Bovine Serum (FBS; Gibco, ThermoFisher, USA) prior to AM isolation. Protein permeability was calculated as a ratio of BAL: serum protein following protein quantification (Pierce™ BCA protein assay).

### Human lung tissue collection and processing

The Lung Tissue Resection study recruited adult patients who were scheduled to have surgery to remove lung tissue as part of their clinical treatment plan (predominantly a lobectomy for lung cancer) at the Thoracic Surgery Unit in Birmingham Heartlands Hospital from September 2017 to July 2019 (REC 17/WM/0272). The aim of this study was to collect human lung tissue samples which were surplus to histopathological requirements following planned thoracic surgery. Lung resections samples were immediately examined in theatre by an experienced member of the surgical team. A section of the sample distant from any tumour (if present), with no evidence of macroscopic pathology, and which was not required for histopathological purposes, was immediately immersed in sterile 0.9% saline. Samples were transported on ice to the laboratory for processing. The airways of lung resection samples were immediately lavaged through with 500 – 2000 mls of sterile 0.9% saline (Baxter, UK) using a 14 gauge needle (Vasofix^®^, Braun, Germany). The lavage fluid was centrifuged at 4°C and 560 g for 10 minutes (Eppendorf AG 5810R, Germany) and the supernatant discarded. The cell pellets were then pooled and re-suspended in 10 mls of RPMI 1640 media containing 10% FBS prior to AM isolation.

### Isolation of human alveolar macrophages

Re-suspended cell pellets from BAL and lung resection lavage were treated identically from this point onwards to isolate AMs. Mononuclear cells were separated by gradient centrifugation using Lymphoprep™ (StemCell Technologies, Vancouver, Canada) as per manufacturer’s instructions. Isolated AMs were washed in PBS and purity assessed by cytospin (25). AMs were re-suspended in RPMI 1640 media supplemented with 10% FBS, 100 U/ml penicillin, 100 µg/ml streptomycin and 2 mM L-glutamine (Sigma-Aldrich) and plated at 5 x 10^5^ per well in a 6-well flat bottom culture plate for HSD-1 functional assays. AMs were cultured in an incubator at 37°C with 5% CO_2_ and media was changed after 24 hours to remove non-adherent cells. For AMs derived from lung tissue resections, flow cytometric staining with CD68 (APC-conjugated mouse anti-human CD68, clone FA-11, Biolegend U.K.) was undertaken to confirm a pure population of AMs. If there was greater than 2% contamination of non-AM cells including interstitial macrophages, the sample was not utilised.

### Quantification of human RAGE

Human ELISA Quantikine kits (R&D Systems) were used to measure concentrations of Receptor for Advanced Glycation End-products (RAGE) in patient BAL as per manufacturer’s instructions.

### Liquid chromatography tandem mass spectrometry for steroid quantification of human serum and broncho-alveolar fluid

Cortisol and cortisone were quantified in serum and BAL at the Steroid Metabolome Core at the University of Birmingham. Briefly, the steroids were extracted from 400 µl of serum or BAL *via* liquid/liquid extraction with 2 ml of MTBE (tert-methyl butyl ether) following addition of the internal standards cortisol-d4 and cortisone-d7 (20 µl of 1000 ng/ml internal standard solution prepared in deuterated methanol). Samples were vortexed and left to equilibrate for 30 minutes. The MTBE layer was then removed and dried under nitrogen at 55°C, this was then reconstituted in 200 µl of methanol: water 50:50 and analysed on a liquid chromatography tandem mass spectrometer, as described previously (26–28). The raw data is available at the following online repository: 10.6084/m9.figshare.22043693.

### Thin layer chromatography assay to assess HSD-1 activity in human alveolar macrophages and mouse lung tissue

This assay was developed and has previously been validated by the Institute for Metabolism and Systems Research at the University of Birmingham (29, 30).

For human AMs, 11β-HSD-1 oxo-reductase activity (conversion of cortisone to cortisol) was determined in adherent cultures containing 500,000 cells incubated in RPMI1640 medium (ThermoFisher Scientific, UK) containing cortisone (100 nmol/l) along with tracer amounts of tritiated cortisone (Perkin Elmer, Beaconsfield, UK) at 37°C and 5% CO_2_ for 6 - 12 hours (29, 30). Steroids were extracted in dichloromethane and separated by thin‐layer chromatography with ethanol/chloroform (8:92) as the mobile phase. Thin‐layer chromatography plates were analysed with a Bioscan imager (Bioscan, Washington, DC, USA), and the fractional conversion of steroids was calculated. HSD-1 activity is expressed in pM/hr/million cells to allow direct comparisons. Intra-assay CV was calculated as 3.6%. Inter-assay CV was calculated from control conditions over 6 experiments as 8.4%.

An equivalent methodology was utilised to measure HSD-1 oxo-reductase activity in murine *ex-vivo* lung tissue as previously described (12). Murine lung tissue was incubated with 100 nmol/l of 11-dehydrocorticosterone (11-DHC) and tritiated [3H] tracer. As above, steroids were extracted and separated before steroid conversion was measured using a Bioscan imager and fractional conversion calculated. Experiments were performed in triplicate, and enzymatic activity is reported as pmol product per mg of tissue per hour.

### Mice

All procedures were performed in compliance with UK law under the Animal [Scientific Procedures] Act 1986. The UK Home Office project licence code was PAAB1C3B2. The 3Rs principles (Reduction, Replacement and Refinement) guided the design and methodology of our animal studies. Male wild-type (WT) C57BL/6 mice were obtained from Harlan UK Limited, Oxford, UK and maintained at the Biomedical Services Unit (BMSU), University of Birmingham, UK. A colony of 11β-Hydroxysteroid Dehydrogenase Type 1 Knockout (HSD-1 KO) C57BL/6 mice was also maintained at BMSU. The breeding pairs for this colony were a kind gift from Professor Gareth Lavery, University of Birmingham (31, 32).

### Intra-tracheal lipopolysaccharide instillation mouse model

Intra-tracheal lipopolysaccharide instillation (IT-LPS) was performed on HSD-1 KO and WT mice aged 8-12 weeks as previously described (33, 34). Briefly, mice were anaesthetised with intraperitoneal injections of metetomidine (60 mg/kg) and ketamine (10 mg/kg). A polyethylene catheter (external diameter 0.61 mm and internal diameter 0.28 mm) was passed into the trachea *via* the mouth under direct visualisation of the vocal cords. Fifty micrograms lipopolysaccharide (LPS, Source Biosciences, UK) in 50 µl sterile PBS, or 50 µl sterile PBS alone, were instilled into the trachea. To reverse the metetomidine, mice were administered with 0.1 ml atipamezole intraperitoneally. Mice were hydrated with two 0.5 ml subcutaneous saline injections, immediately and at 6 hours post-procedure. Following IT instillations, the hair around the neck of each mouse was removed using Veet (Unilever, UK); arterial oxygen saturations were monitored using infrared pulse oximetry (MouseOx Plus, Starr Life Sciences Corp, USA) in accordance with manufacturer’s instructions as previously described (34). Forty eight hours post IT-LPS, mice were deeply anaesthetised with 5% isoflurane in oxygen delivered at 1.5 l/min. Cardiac puncture was performed and death confirmed. The 48 hour timepoint was chosen following optimisation studies which revealed that peak cellular inflammation and BAL inflammatory cytokine concentrations (tumour necrosis factor-α, interleukin-6 and interleukin-1b) were observed at this timepoint (34). Immediately post-mortem, BAL fluid was collected by lavaging the lungs twice with 600 μl of PBS/1% EDTA *via* a tracheal tube.

### Caecal ligation and puncture mouse model

Caecal ligation and puncture was performed on HSD-1 KO and WT mice aged 8-12 weeks as previously described (35). Briefly, subcutaneous buprenorphine (0.1 mg/kg body weight) was administered 15 minutes prior to the procedure. Mice were anaesthetised with 5% isoflurane gas in oxygen delivered at 1.5 l/min for induction, then at 1-3% isoflurane for maintenance anaesthesia. All surgery was performed with aseptic technique. Midline laparotomy was performed followed by exposure of the caecum, ligation of the lower 30% with 2.0 nylon suture (Ethicon, UK) and single puncture of the ligated caecum with a 19G microlance needle (BD, UK). A small amount of faeces was expressed by compressing the ligated caecum with forceps prior to being placed back into the abdomen and closed with 6.0 Vicryl^®^ (Ethicon, UK). Skin closure was with 4.0 Prolene^®^ (Ethicon, UK). Surgery was performed on heated tables set at 37.5°C. All animals recovered in heat boxes and recovery incubators until euthanised. Immediate post-operative hydration was with 0.5mls of Hartmann’s solution (Aqupharm 11^®^) and another dose of buprenorphine. At 6 hours post-surgery mice were assessed and another 0.5 mls of subcutaneous fluid was administered. Mice were then euthanised at 16 hours post-surgery. Sham surgery was identical except for the lack of caecal ligation and puncture after externalisation of the caecum from the abdomen. Due to the severity of this model, our UK Home Office approved project licence limited the duration of the CLP model to a maximum of 24 hours and prohibited use of mortality as an endpoint in this model. We used a timepoint of 16 hours as this had previously demonstrated a neutrophilic infiltrate within the peritoneum whilst limiting the duration of animal suffering. Samples of peritoneal lavage fluid (PLF) were collected immediately post-mortem by instilling 1 ml of PBS/1% EDTA into both upper quadrants of the abdomen and subsequently aspirating from both lower quadrants.

### Analysis of murine lavage samples

Broncho-alveolar and peritoneal lavage fluid samples were centrifuged at 400 g for 10 minutes; supernatants were then aspirated and stored at -80°C for cytokine and protein content analysis. The BAL and PLF cell pellets were re-suspended in 1 ml PBS including 2% BSA and 10% murine serum (Sigma-Aldrich), and incubated on ice for 15 minutes to allow blocking of non-specific Fc receptor binding, prior to antibody labelling. Cell pellets from BAL and PLF were assessed for cellular inflammation and apoptotic cell number by flow cytometry (LSR Fortessa X-20, BD Biosciences, UK) using fluorophore-conjugated antibodies (eBioscience). Granulocytes were enumerated by gating on cells with a high forward and high side scatter distribution. Neutrophils were defined as CD11c^-^CD11b^+^Gr1^+^F4/80^-^, monocytes as CD11c^+^CD11b^+^, and F4/80^+^ as macrophages. Apoptosis was analysed as FITC - Annexin V and SyTOX Blue (Invitrogen) double positive populations. BAL RAGE concentrations (DuoSet ELISA, R&D systems, UK), and cytokine concentrations (Luminex array, R&D systems, UK) were measured using BAL and PLF supernatants. The following cytokines were measured: Interleukin (IL)-1β, IL-6, tumour necrosis factor-alpha (TNFα), keratinocyte chemoattractant (KC) and vascular endothelial growth factor (VEGF). Protein permeability index was calculated as a ratio of BAL or PLF protein to serum protein. Protein concentrations in biofluids were measured using a commercial kit (Pierce™ BCA protein assay).

PLF was diluted serially and incubated at 37°C in pre-prepared Lysogeny broth (LB-Lennox, Merck) agar plates for 24 hours; bacterial colony forming units (CFU) were then counted and CFU/ml calculated from the original dilutions.

### Murine lavage antibody staining panel

**Table:** 

Antibody	Clone	Fluorophore	Manufacturer	Isotype Control	Dilution
Anti-mouse F4/80	T45-2342	PE	BD Biosciences	Rat, IgG2Aκ	1: 100
Anti-mouse CD11c	HL3	PE-Cy7	BD Biosciences	Hamster, IgG1 λ1	1: 100
Anti-mouse CD11b	M1/70	APC	BD Biosciences	Rat, IgG2Bκ	1: 100
Anti-mouse Ly6G (Gr1)	1A8	APC-Cy7	BD Biosciences	Rat, IgG2Aκ	1: 100

APC, Allophycocyanin; PE, Phycoerythrin.

### Statistical analysis

Data were analysed using Prism 9 software (GraphPad, USA). Normality of data was assessed using the D’Agostino & Pearson test. Differences between 2 non-parametric data sets was assessed using Mann-Whitney tests. Differences between 3 non-parametric data sets was assessed using Kruskal-Wallis test followed by Dunn’s multiple comparisons tests. Two-tailed p-values of ≤0.05 were considered as significant. Results from parametric data are shown as mean and standard deviation. Results from non-parametric data are shown as median and interquartile range. Monotonic associations between non-parametric variables were assessed using Spearman’s rank-order correlation coefficient.

## Results

### Patient characteristics

Demographics and physiological characteristics of ICU patients from both the BALTI-2 sub-study and the AM-ARDS study are shown in **Table 1**. Of the ARDS patients recruited to the BALTI-2 sub-study, 9 received intravenous salbutamol and 11 received placebo as part of the clinical trial. Alveolar macrophages (AMs) were also isolated from the lung tissue of 21 patients who underwent lobectomy (mean yield of 9 million AMs per patient). The mean age of lobectomy patients was 68 years (SD 6.9 years). The male: female split for lobectomy patients was 12:9. For the 4 healthy volunteers, the mean age was 22 years (SD 1.7 years) and the male: female split was 1:3.

**Table 1:** ICU Patient Demographics and Physiological Characteristics.

	BALTI-2 sub-study		AM-ARDS Study	
	Sepsis with ARDS (n=20)	Sepsis without ARDS (n=5)	Sepsis with ARDS (n=21)	Sepsis without ARDS (n=17)
**Age in years: mean (SD)**	60.3 (18.2)	65.2 (13.3)	59.2 (13.9)	55.1 (16.3)
**Male Sex: n (%)**	12 (60%)	3 (60%)	15 (71%)	11 (65%)
**SOFA score:** **mean (SD)**	11.1 (2.1)	10.6 (2.6)	12.5 (3.8)	10.3 (2.7)
**APACHE-II score:** **mean (SD)**	24.7 (5.4)	24.8 (5.0)	18.6 (5.5)	15.2 (5.8)
**Murray Lung Injury Score: mean (SD)**	2.78 (0.42)	1.13 (0.77)	2.57 (0.50)	2.13 (0.46)
**30 day mortality: n (%)**	12 (60%)*	1 (20%)	8 (38.1%)	3 (17.6%)
**BAL leukocyte count x10^6^: median (IQR)**	32.2 (17.8 – 44.8)	9.0	15.8 (7.4 – 31.3)	6.4 (3.8 – 27.0)
**PMNs in BAL:** **% mean (SD)**	68.0 (19.0)	6	69.9 (21.5)	49.0 (30.6)

*Of the ARDS patients in the BALTI-2 sub-study, those receiving intravenous salbutamol had a 56% mortality and those receiving placebo had a 64% mortality at 30 days. APACHE-II: Acute Physiology and Chronic Health Evaluation II. BAL, Broncho-alveolar lavage; ICU, Intensive care unit; IQR, Inter-quartile range; PMN, Polymorphonuclear cell; SD, Standard deviation; SOFA, Sepsis-related Organ Failure Assessment.

### Serum and BAL glucocorticoid ratios in sepsis patients with and without ARDS

**Figure 1:**
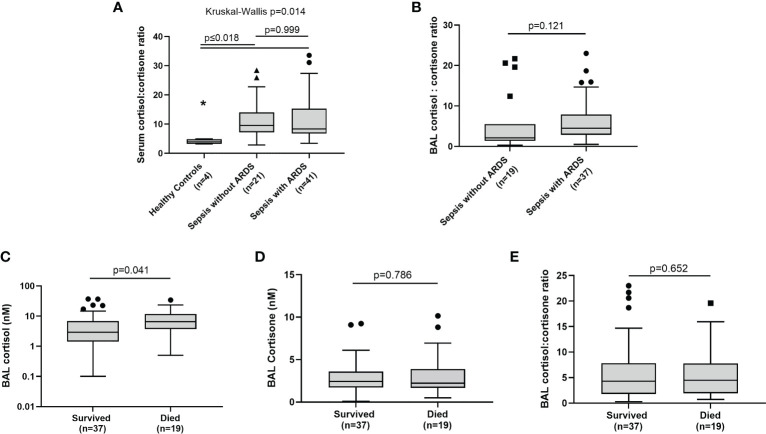
BAL and serum glucocorticoid ratios in sepsis patients with and without ARDS. Combined data from the AM-ARDS and BALTI-2 sub-study shown, n-56. Healthy volunteers n=4. Data shown as Tukey’s box plots. **(A)** Serum cortisol: cortisone ratios in healthy volunteers, sepsis patients with ARDS and sepsis patients without ARDS. (medians 3.9 v 9.59 v 8.30, Kruskal Wallis p=0.014). **(B)** BAL cortisol: cortisone ratios in sepsis patients with and without ARDS (2.10 v 4.50, p=0.121). **(C)** Association between BAL cortisol and 30 day mortality following ICU admission, across all sepsis patients (with and without ARDS) (medians 6.56 v 2.90 nM, p=0.041). **(D)** Association between BAL cortisone and 30 day mortality across all sepsis patients, with and without ARDS (2.43 v 2.22 nM, p=0.786). **(E)** Association between BAL cortisol: cortisone ratio and 30 day mortality across all sepsis patients, with and without ARDS (4.30 v 4.50, p=0.652). BAL, Broncho-alveolar lavage IT-PBS; Intra-tracheal phosphate buffered saline. IT-LPS; Intra-tracheal lipopolysaccharide.

To investigate global HSD-1 reductase activity levels across sepsis patients with and without ARDS, we examined cortisol: cortisone ratios in the BAL and serum of patients recruited to the BALTI-2 sub-study and AM-ARDS studies; data from these two studies were then combined. We also analysed serum steroid concentrations and ratios in healthy volunteers. There was no difference in serum cortisol and cortisone concentrations between healthy controls, sepsis patients without ARDS and sepsis patients with ARDS (data not shown). Serum cortisol: cortisone ratios were lower in healthy volunteers compared to both groups of sepsis patients (**Figure 1A**, 3.9 v 9.59 and 8.30, p ≤ 0.018). However, there was no difference in serum cortisol: cortisone ratios between sepsis patients with and without ARDS (**Figure 1A**, medians 8.30 v 9.59 p=0.999). There was also no difference in BAL cortisol: cortisone ratios between sepsis patients with and without ARDS (**Figure 1B**, medians 4.5 v 2.1, p=0.121). There was no difference in BAL cortisol and cortisone concentrations between sepsis patients with and without ARDS (data not shown). Combined data across all sepsis patients (with and without ARDS), showed that BAL cortisol levels were elevated in patients who died within 30 days of ICU admission, compared to survivors (**Figure 1C**: medians 6.56 vs 2.90 nM, p=0.041). However, across all sepsis patients (with and without ARDS) there was no association between BAL cortisone concentrations and 30 day mortality (**Figure 1D**) or BAL cortisol: cortisone ratio and 30 day mortality (**Figure 1E**).

### Alveolar Macrophage HSD-1 reductase activity in sepsis patients with and without ARDS

**Figure 2:**
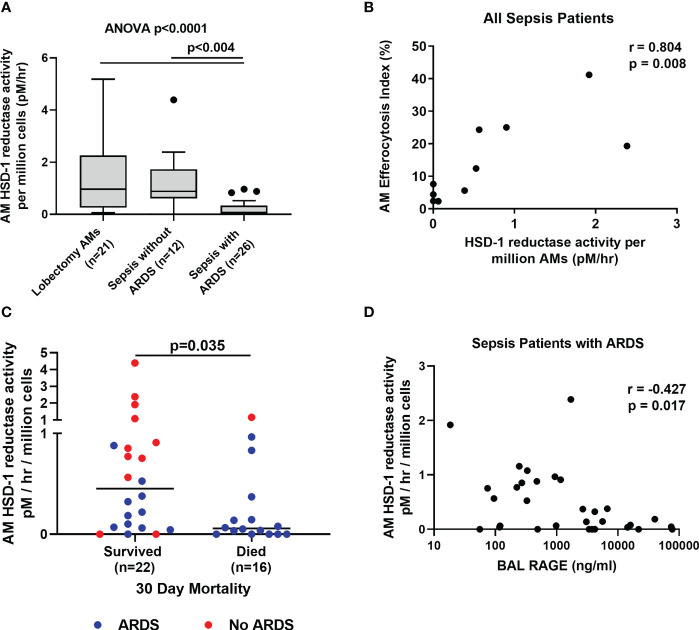
Alveolar macrophage HSD-1 reductase activity compared between patient groups. **(A)** AM HSD-1 reductase activity in lobectomy patients, sepsis patients without ARDS and sepsis patients with ARDS (medians 0.967 v 0.882 v 0.075 pM/hr/10^6^ AMs, Kruskal-Wallis p<0.0001, Dunn’s *post-hoc* p<0.004). Data shown as Tukey’s box plots. **(B)** Correlation between AM HSD-1 reductase activity and alveolar macrophage efferocytosis index, across all sepsis patients with and without ARDS (r= 0.804, p=0.008, n=10). **(C)** Association between AM HSD-1 reductase activity and 30 day mortality following ICU admission across all sepsis patients with and without ARDS (medians 0.453 v 0.052 pM/hr/10^6^ AMs, p=0.035). **(D)** Correlation between AM HSD-1 reductase activity and BAL RAGE concentrations in sepsis patients with ARDS (r= -0.487, p=0.022, n=22). AM, Alveolar Macrophage; BAL, Broncho-alveolar lavage; HSD-1, 11β Hydroxysteroid Dehydrogenase Type 1; RAGE, Receptor for Advanced Glycation End Products.

Local autocrine HSD-1 reductase activity in macrophages has been shown to be functionally important (12). This activity is thought to make a relatively small contribution to total circulating cortisol and cortisone levels, but self-regulates macrophage function *via* autocrine signalling. Therefore, we investigated HSD-1 reductase activity within AMs from sepsis patients with and without ARDS. AM HSD-1 reductase activity is impaired in sepsis patients with ARDS compared to sepsis patients without ARDS and lobectomy patients (**Figure 2A**, (medians 0.967 v 0.882 v 0.075 pM/hr/10^6^ AMs, p<0.004). Across all sepsis patients (with and without ARDS), AM HSD-1 reductase activity positively correlated with AM efferocytosis (**Figure 2B**, r=0.804, p=0.008). Efferocytosis describes the ability of a macrophage to clear apoptotic cells. Combined data across all sepsis patients (with and without ARDS) shows that AM HSD-1 reductase activity was significantly lower in patients who died within 30 days of ICU admission, compared to those who survived (**Figure 2C**, medians 0.052 v 0.453 pM/hr/10^6^ AMs, p=0.035). Fifteen of the 16 sepsis patients who died within 30 days of ICU admission had ARDS. In sepsis patients with ARDS, a trend towards lower AM HSD-1 reductase activity was observed in patients who died within 30 days of ICU admission compared to those who survived, however this did not reach statistical significance (data not shown, medians 0.052 v 0.185 pM/hr/10^6^ AMs, p=0.122). In sepsis patients with ARDS, a negative correlation was observed between AM HSD-1 reductase activity and BAL RAGE concentrations (**Figure 2D**, r=-0.427, p=0.017). RAGE is a biomarker for alveolar epithelial cell injury in ARDS (36).

### HSD-1 KO mice develop greater pulmonary inflammation following IT-LPS

**Figure 3:**
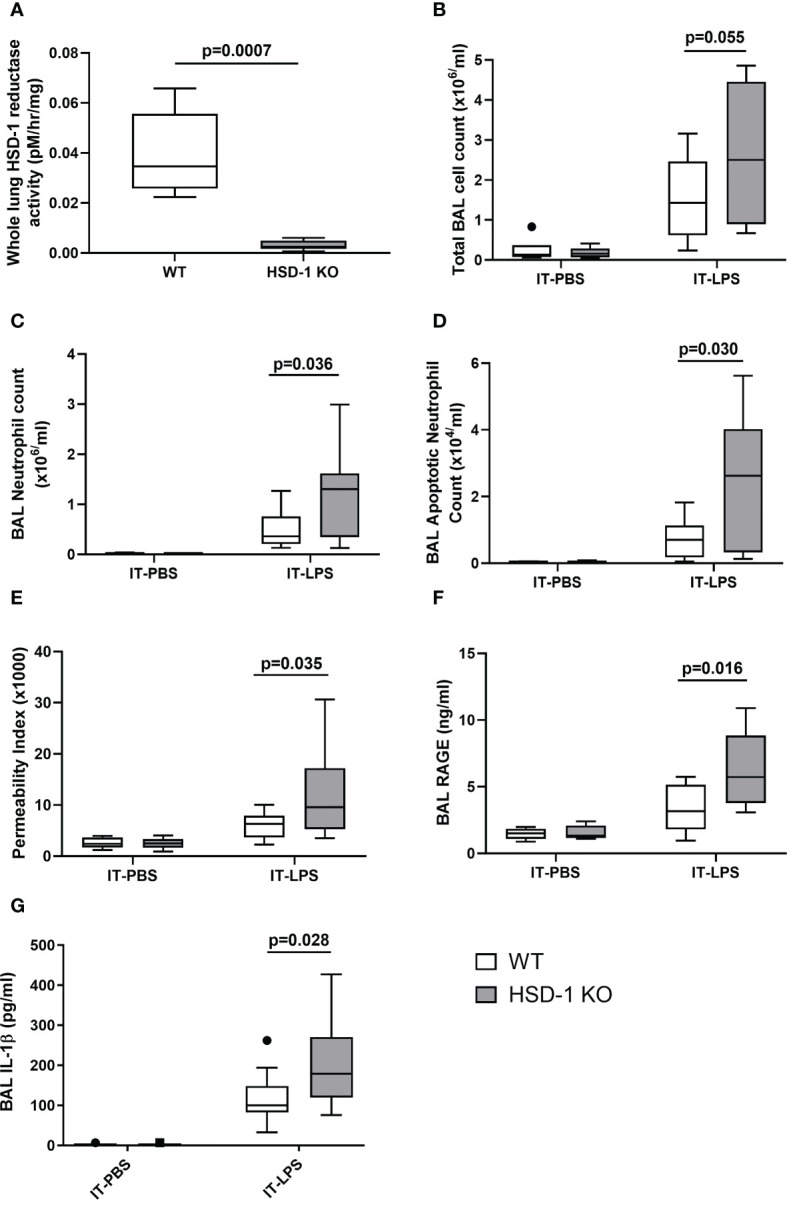
IT-LPS injury in HSD-1 knockout mice is associated with increased alveolar neutrophil infiltration, alveolar permeability and RAGE concentrations. Time course of IT-PBS and IT-LPS injury was 48 hours. For IT-LPS, n≥10 each group. For IT-PBS, n=6 per group. Data shown as Tukey’s box plots. **(A)** Whole lung HSD-1 reductase activity in uninjured WT and HSD-1 KO mice (medians 0.0345 vs 0.0025 pM/hr/mg, p=0.0007, n≥5 per group). **(B)** BAL total cell count in HSD-1 KO and WT (IT-LPS treated medians 2.50 v 1.43 x10^6^/ml, p=0.055). **(C)** BAL neutrophil counts in HSD-1 KO and WT mice (IT-LPS treated medians 1.30 v 0.362 x10^6^/ml, p=0.036). **(D)** BAL apoptotic neutrophil counts in HSD-1 KO and c WT mice (IT-LPS treated medians 2.62 v 0.701 x10^4^/ml, p=0.030). **(E)** Alveolar protein permeability in HSD-1 KO and WT mice (IT-LPS treated medians 9.55 v 6.30 x10^3^, p=0.035). **(F)** Concentrations of BAL RAGE in HSD-1 KO and WT mice (IT-LPS treated medians 5.74 v 3.17 ng/ml, p=0.016). **(G)** Concentrations of BAL IL-1β in HSD-1 KO and WT mice (IT-LPS treated medians 179 v 100 pg/ml, p=0.028). BAL, Broncho-alveolar lavage; HSD-1, 11β Hydroxysteroid Dehydrogenase Type 1; IT-LPS, Intra-Tracheal Lipopolysaccharide; IT-PBS, Intra-Tracheal Phosphate Buffered Saline; KO, Knockout; RAGE, Receptor for Advanced Glycation End Products; WT, Wild Type.

To investigate the functional consequences of reduced AM HSD-1 reductase activity that we observed in patients with ARDS, the IT-LPS model of acute inflammatory lung injury was utilised in HSD-1 knockout (KO) and wild type (WT) mice. Validation studies showed that uninjured HSD-1 KO mice had negligible whole lung HSD-1 reductase activity compared to uninjured WT mice (**Figure 3A**: medians 0.0345 vs 0.0025 pM/hr/mg, p=0.0007). Previous studies also showed a loss of HSD-1 reductase activity in macrophages derived from HSD-1 KO mice (12). At 48 hours following IT-LPS injury, a trend towards an increased total BAL cell count was observed in HSD-1 KO mice compared to WT mice, but did not reach statistical significance (**Figure 3B**, medians 2.50 v 1.43 x10^6^/ml, p=0.055). Elevated BAL neutrophil counts (**Figure 3C**, medians 1.30 v 0.362 x10^6^/ml, p=0.036) and apoptotic neutrophil counts (**Figure 3D**: medians 2.62 v 0.701 x10^4^/ml, p=0.030) were observed in HSD-1 KO mice compared to WT mice following IT-LPS. Alveolar protein permeability was also elevated in HSD-1 KO mice compared to WT mice following IT-LPS (**Figure 3E**, medians 9.55 v 6.30 x10^3^, p=0.035). Concentrations of BAL RAGE (**Figure 3F**, medians 5.74 v 3.17 ng/ml, p=0.016) and IL-1β (**Figure 3G**, medians 179 v 100 pg/ml, p=0.028) were elevated in HSD-1 KO mice, compared to WT mice following IT-LPS. No difference in BAL cytokines TNFα, IL-6, KC and VEGF were observed between HSD-1 KO and WT mice following IT-LPS injury (data not shown, p>0.05 for all). No difference in arterial oxygen saturations was observed between HSD-1 KO and WT mice following IT-LPS injury (data not shown, p>0.05). Following IT-PBS, no difference was observed in total BAL cell count, BAL neutrophil count, BAL apoptotic neutrophil count, alveolar protein permeability, BAL RAGE or BAL IL-1β between WT and HSD-1 KO mice (**Figures 3A-G**, p>0.05). Thus, the IT-LPS model in HSD-1 KO mice recapitulates some key features of human sepsis-related ARDS including development of a highly neutrophilic alveolar infiltrate, elevated BAL RAGE concentrations and increased alveolar protein permeability.

### HSD-1 KO mice develop accumulation of apoptotic neutrophils following CLP

**Figure 4:**
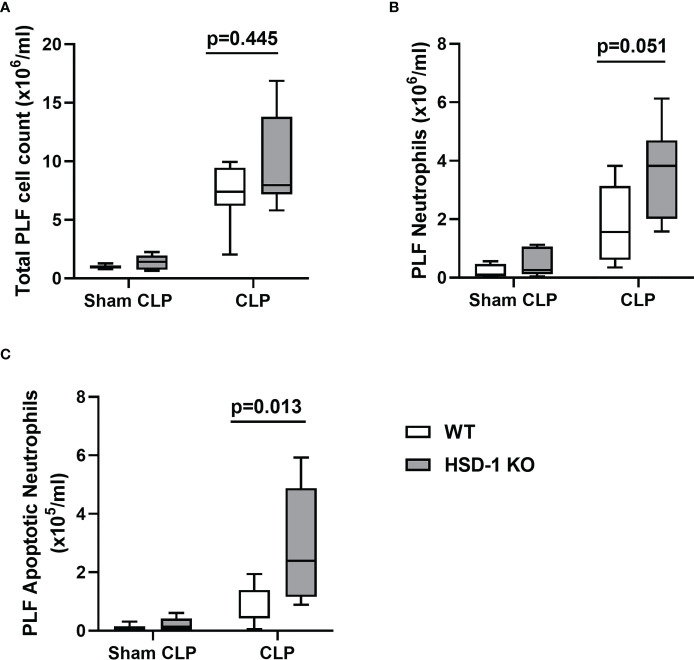
CLP injury in HSD-1 knockout mice is associated with accumulation of apoptotic neutrophils within the peritoneum. Time course of CLP injury and sham CLP was 16 hours, n≥6 in each group (for both CLP and sham CLP). **(A)** PLF total cell count in HSD-1 KO and WT mice (CLP medians 4.06 v 5.61 x10^6^, p=0.445). **(B)** PLF neutrophil count in HSD-1 KO and WT mice (CLP medians 3.83 v 1.57 x10^6^/ml, p=0.051). **(C)** PLF apoptotic neutrophil count in HSD-1 KO and WT mice (CLP medians 2.40 v 0.472 x10^5^/ml, p=0.013). CLP, Caecal ligation and puncture; HSD-1, 11β Hydroxysteroid Dehydrogenase Type 1; KO, Knockout; PLF, Peritoneal Lavage Fluid; WT, Wild Type.

To investigate the impact of HSD-1 deficiency on sepsis, the CLP model of peritoneal sepsis was utilised in HSD-1 KO and WT mice. At 16 hours following CLP injury, there was no difference in total PLF cell count observed between HSD-1 KO and WT mice (**Figure 4A**, p=0.445). A trend towards an increased PLF neutrophil count was observed in HSD-1 KO mice compared to WT mice, but did not reach statistical significance (**Figure 4B**, 3.83 v 1.57 x10^6^/ml, p=0.051). However, the PLF apoptotic neutrophil count was elevated in HSD-1 KO mice compared to WT mice following CLP injury (**Figure 4C**, medians 2.40 v 0.472 x10^5^/ml, p=0.013). No difference in PLF cytokines TNFα, IL-1β, IL-6, and KC were observed between HSD-1 KO and WT mice following CLP injury (data not shown, p>0.05 for all). No difference in peritoneal protein permeability index was observed between HSD-1 KO and WT mice following CLP injury (data not shown, p>0.05). Following sham CLP, no difference was observed in total PLF cell count, PLF neutrophil count or PLF apoptotic neutrophil count between WT and HSD-1 KO mice (**Figures 4A-C**, p>0.05).

## Discussion

In this study, using the ratio of cortisol: cortisone as an indicator of HSD enzymatic activity, we found that total HSD-1 activity does not differ significantly between sepsis patients with and without ARDS. However, we found for the first time that AM HSD-1 reductase activity is impaired in patients with sepsis-related ARDS, compared to sepsis patients without ARDS. Across all critically ill sepsis patients (with and without ARDS), impaired AM HSD-1 reductase activity was associated with defective efferocytosis and increased 30-day mortality; in contrast, total HSD-1 activity was not associated with mortality. We also found that impaired AM HSD-1 reductase activity is associated with elevated BAL RAGE concentrations (a marker of alveolar epithelial injury) in patients with sepsis-related ARDS.

Changes in AM HSD-1 reductase activity make a relatively small contribution to serum and total BAL cortisol and cortisone levels, indicating that these paracrine and endocrine glucocorticoid ratios are predominantly determined by HSD-1 activity at the organ level (i.e. liver, combined whole lung). The association between AM HSD-1 reductase activity and mortality may be explained by the ability of HSD-1 reductase to regulate autocrine intracellular cortisol levels in AMs, thereby amplifying the ability of local glucocorticoids to exert their anti-inflammatory effects. Despite abundant substrate in sepsis-related ARDS, the loss of HSD-1 autocrine steroid amplification in AMs renders these cells less sensitive to the anti-inflammatory effects of both endogenous and exogenous local alveolar glucocorticoids. This leads to the impaired AM efferocytosis, increased BAL RAGE concentrations, inflammatory injury and mortality observed. Our findings are supported by a previous murine study in which myeloid-specific deletion of HSD-1 diminished the anti-inflammatory response of macrophages to exogenous glucocorticoid treatment, leading to greater inflammatory injury. However, the circulating endocrine glucocorticoid levels in myeloid-specific HSD-1 KO mice were unchanged compared to WT mice, thus indicating that the increased inflammation could be attributed to impaired autocrine glucocorticoid amplification in myeloid cells (12).

Our data suggest a positive association between AM HSD-1 reductase activity and efferocytosis. Efferocytosis describes the ability of a macrophage to clear apoptotic cells. The ability of glucocorticoids to upregulate AM efferocytosis is dependent on HSD-1 expression (17, 18). Glucocorticoids increase macrophage efferocytosis partly by upregulating expression of the efferocytosis receptor Mer (37, 38) and downregulating expression of the inhibitory receptor Signal Regulatory Protein-α (SIRPα) (39) on the surface of macrophages. Thus, the impaired AM efferocytosis we observe in patients with sepsis-related ARDS may be secondary to impaired HSD-1 reductase autocrine signalling. These results, together with our previous findings (14), indicate that AMs in sepsis-related ARDS have a dysfunctional phenotype with impaired HSD-1 reductase activity and impaired efferocytosis. Without efficient clearance, persistent apoptotic neutrophils undergo secondary necrosis (40, 41), releasing further pro-inflammatory factors into the extracellular space, likely injuring the alveolar epithelium and causing the observed increase in BAL RAGE concentrations, protein permeability and mortality.

The IT-LPS model of inflammatory lung injury and CLP model of peritoneal sepsis in HSD-1 KO mice support these findings by demonstrating increased accumulation of apoptotic neutrophils. The IT-LPS model also demonstrates increased alveolar protein permeability and BAL RAGE concentrations (both soluble markers of alveolar epithelial damage) in HSD-1 KO mice (36, 42). Our findings are in keeping with those from previous studies showing that HSD-1 KO mice undergo greater acute inflammatory injury (7). These findings also support the association between HSD-1 deficiency and impaired macrophage efferocytosis, which leads to the accumulation of apoptotic neutrophils in these models.

Previous studies have shown that pro-inflammatory cytokines promote HSD-1 expression (43); however, despite a highly inflammatory alveolar microenvironment in ARDS, AM HSD-1 remains suppressed. This paradoxical finding suggests the presence of a global AM dysfunction in ARDS patients, with impaired HSD-1 autocrine signalling and efferocytosis. A global dysfunction such as this may be driven by a shift in AM metabolic profile (44); further studies are required to determine whether there is evidence of AM metabolic reprogramming in sepsis-related ARDS.

These findings lead to questions regarding the utility of therapeutic glucocorticoids in patients with ARDS, especially in those patients without SARS-CoV-2 infection. In the 1980s, trials of high-dose methylprednisolone (30 mg/kg) in ARDS had clear adverse outcomes in patients, demonstrating increased mortality (45, 46). Other studies during that period showed no differences with methylprednisolone therapy (47, 48).In the 2000s, trials focussed on using a moderate dose of intravenous methylprednisolone (initially 1-2 mg/kg) with tapering down over several weeks. These studies showed a reduction in duration of mechanical ventilation and during of ICU stay, but no effect on mortality in ARDS patients (49, 50). However, early hydrocortisone therapy in patients with severe influenza A/H1N1 induced ARDS led to increased mortality, more secondary infections, and a trend towards longer duration of invasive ventilation (51). The 2019 Faculty of Intensive Care Medicine/Intensive Care Society guidelines (52) on the management of ARDS assessed four meta-analyses of corticosteroid treatment for ARDS. This pre-pandemic guideline concluded that the evidence to support use of corticosteroids in ARDS was of low to very low quality, and that a suitably powered multicentre randomised controlled trial with long term follow up was required. A subsequently published multicentre trial of dexamethasone in non-COVID patients with moderate-severe ARDS reported a reduction in duration of invasive ventilation and mortality (53).

As part of the RECOVERY trial undertaken during the COVID-19 pandemic, use of dexamethasone reduced mortality for those patients with SARS-CoV-2 pneumonitis requiring oxygen or invasive ventilation (54). The greatest treatment benefit was observed in those patients receiving invasive ventilation, of whom the vast majority would have met the criteria for ARDS. Latent class analysis subsequently showed that a ‘hyper-inflammatory’ subgroup of patients with COVID-19-related ARDS had improved survival with corticosteroid therapy, whereas the ‘hypo-inflammatory’ subgroup did not (55). Part of the reason for the differential response to corticosteroids between phenotypic subgroups of ARDS may be related to AM HSD-1 expression. Single-cell transcriptomic analysis of BAL macrophages in patients with COVID-19-related ARDS reveals that a ficolin-1^+^/osteopontin^+^ subpopulation of macrophages appear to play a pathogenic role (56). This macrophage subset was associated with greater disease severity, impaired transcription of ligands which facilitate efferocytosis (Protein S), and promotion of inflammatory monocyte and neutrophil phenotypes. A pro-resolving subset of AMs expressing fatty acid-binding protein 4 was also identified. Differential expression of HSD-1 between these macrophage subsets may contribute to their respective pathogenic and pro-resolving phenotype and function. Further studies are required to determine whether HSD-1 expression varies between hyper- and hypo-inflammatory subgroups of ARDS patients, and also between AM subsets within individual ARDS patients.

Part of the reason that therapeutic corticosteroids fail to improve clinical outcomes in a significant proportion of all sepsis-related ARDS patients may be due to impairment of local AM HSD-1 autocrine signalling. Therefore, strategies to upregulate AM HSD-1 reductase activity and restore autocrine signalling in ARDS patients could enable both endogenous and exogenous glucocorticoids to exert their anti-inflammatory effects locally. AM efferocytosis function would be restored, thereby reducing secondary necrosis of apoptotic neutrophils. Strategies to achieve this include HSD-1 gene therapy targeted at alveolar macrophages *via* viral vector or *ex-vivo* modification (57). Further studies are required to determine the feasibility of these approaches.

This study has some limitations. The differences in 30-day mortality and APACHE-II score between the two clinical cohorts reflect clinical advances made in ARDS patient management during this time. The advent of lung protective ventilation including use of lower tidal volumes (58), conservative fluid management (59), and prone positioning (60) all became standard of care for ARDS management in the time period between the two cohorts, and likely account for the improved mortality and reduced severity observed in the AM-ARDS cohort. However, these improvements mean that our study is limited by heterogenous clinical outcomes between patient cohorts. Also, the definitions of ARDS differed slightly between the AM-ARDS study (Berlin) and BALTI-2 sub-study (AECC) again due to the temporal gap between these studies. A limitation of sampling was that ARDS patient BALs were often highly neutrophilic, making AM isolation challenging. The average number of AMs isolated from BAL was 1 million, thus often only one functional assay could be performed; measurement of both AM HSD-1 reductase activity and efferocytosis could only be undertaken in a minority of patients. A limitation of the mouse studies was the use of global HSD-1 knockout mice as opposed to myeloid-specific HSD-1 knockouts.

In conclusion, whilst AM HSD-1 reductase activity does not shape total BAL or serum cortisol: cortisone ratios, it likely plays an important autocrine role in regulating AM function by locally amplifying the anti-inflammatory activity of glucocorticoids. The loss of HSD-1 steroid amplification renders AMs less sensitive to BAL glucocorticoids, leading to the impaired AM efferocytosis, increased BAL RAGE concentrations and mortality observed in sepsis-related ARDS. The IT-LPS model of lung injury and CLP model of peritoneal sepsis in HSD-1 knockout mice support these findings by demonstrating exaggerated inflammatory injury associated with accumulation of apoptotic neutrophils. Therefore, strategies to upregulate AM HSD-1 expression in patients with sepsis-related ARDS could enable endogenous (and exogenous) glucocorticoids to exert their anti-inflammatory effects locally and restore AM function, thus improving clinical outcomes.

## Data availability statement

The original contributions presented in the study are publicly available. This data can be found here: https://figshare.com/s/815215aae7542d0bbdd0.

## Ethics statement

The studies involving human participants were reviewed and approved by UK REC 06/Q1604/123 for the BALTI-2 sub-study; UK REC 16/WA/0169 for the AM-ARDS study; and UK REC 17/WM/0272 for the Lung Tissue study. The patients/participants or their legal representatives provided their written informed consent to participate in this study. The animal study was reviewed and approved by local ethics committee and UK Home Office (project licence code PAAB1C3B2).

## Author contributions

RM, MM, PS, MC, GP and DT contributed to the study conception and design. RM, SL, CB, AS, DP, RH and BN contributed to data acquisition. RM drafted the manuscript. All authors contributed to the article and approved the submitted version.
