# Supplementary figures and images for: Impaired alveolar macrophage 11β-hydroxysteroid dehydrogenase type 1 reductase activity contributes to increased pulmonary inflammation and mortality in sepsis-related ARDS
Source: Front Immunol. 2023 Apr 27;14:1159831. doi: 10.3389/fimmu.2023.1159831 (PMC10172463; doi:10.3389/fimmu.2023.1159831)

**Clinical Studies: Recruitment and Sampling**

AM-ARDS Study


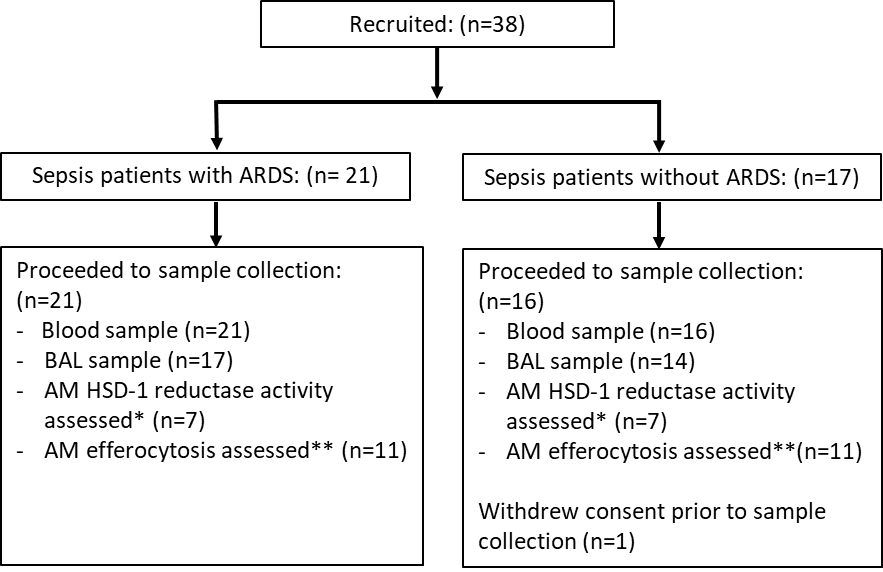


BALTI-2 Sub-Study


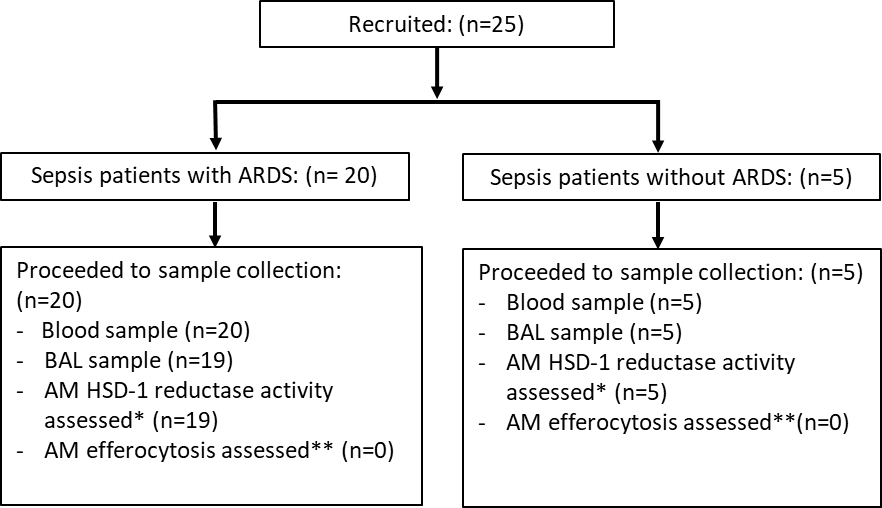

Supplement: Supplementary file 1 [file DataSheet_1.docx]
